# Oral re-vaccination of Eurasian wild boar with Mycobacterium bovis BCG yields a strong protective response against challenge with a field strain

**DOI:** 10.1186/1746-6148-10-96

**Published:** 2014-04-26

**Authors:** Christian Gortazar, Beatriz Beltrán-Beck, Joseba M Garrido, Alicia Aranaz, Iker A Sevilla, Mariana Boadella, Konstantin P Lyashchenko, Ruth C Galindo, Vidal Montoro, Lucas Domínguez, Ramón Juste, Jose de la Fuente

**Affiliations:** 1 SaBio IREC (CSIC-UCLM-JCCM), Ronda de Toledo s/n, 13005 Ciudad Real, Spain; 2 NEIKER-Tecnalia, Animal Health Department, C/Berreaga 1, E-48160 Derio, Bizkaia, Spain; 3 Departamento de Sanidad Animal, Facultad de Veterinaria, Universidad Complutense de Madrid, Avenida de Puerta de Hierro s/n, 28040 Madrid, Spain; 4 Chembio Diagnostic Systems Inc., Medford, NY 11763, USA; 5 Centro de Vigilancia Sanitaria Veterinaria (VISAVET), Facultad de Veterinaria, Universidad Complutense de Madrid, Avenida de Puerta de Hierro s/n, 28040 Madrid, Spain; 6 Department of Veterinary Pathobiology, Center for Veterinary Health Sciences, Oklahoma State University, Stillwater, OK 74078, USA

**Keywords:** Bacillus Camette Guerin, *Sus scrofa*, Tuberculosis, Vaccination and challenge, Wildlife disease control

## Abstract

**Background:**

Field vaccination trials with *Mycobacterium bovis* BCG, an attenuated mutant of *M. bovis*, are ongoing in Spain, where the Eurasian wild boar (*Sus scrofa*) is regarded as the main driver of animal tuberculosis (TB). The oral baiting strategy consists in deploying vaccine baits twice each summer, in order to gain access to a high proportion of wild boar piglets. The aim of this study was to assess the response of wild boar to re-vaccination with BCG and to subsequent challenge with an *M. bovis* field strain.

**Results:**

BCG re-vaccinated wild boar showed reductions of 75.8% in lesion score and 66.9% in culture score, as compared to unvaccinated controls. Only one of nine vaccinated wild boar had a culture-confirmed lung infection, as compared to seven of eight controls. Serum antibody levels were highly variable and did not differ significantly between BCG re-vaccinated wild boar and controls. Gamma IFN levels differed significantly between BCG re-vaccinated wild boar and controls. The mRNA levels for IL-1b, C3 and MUT were significantly higher in vaccinated wild boar when compared to controls after vaccination and decreased after mycobacterial challenge.

**Conclusions:**

Oral re-vaccination of wild boar with BCG yields a strong protective response against challenge with a field strain. Moreover, re-vaccination of wild boar with BCG is not counterproductive. These findings are relevant given that re-vaccination is likely to happen under real (field) conditions.

## Background

Wildlife vaccination is increasingly being explored as a tuberculosis (TB) control tool in all major wildlife reservoirs. An attenuated mutant of *M. bovis, Mycobacterium bovis* BCG (bacille Calmette Guerin [1]), is often the vaccine used in wildlife vaccination trials worldwide [2-8]. In countries such as UK and the Republic of Ireland, Eurasian badger (*Meles meles*) vaccination field trials are becoming part of the TB control strategies [9]. Field vaccination trials are also ongoing in Spain, where the Eurasian wild boar (*Sus scrofa*) is regarded as the main driver of animal TB at the wildlife-livestock interface [10-12].

In Mediterranean Spain, modeling of tuberculosis in wild boar populations predicted that over 70% of the annual cohort of wild boar piglets would be needed to be vaccinated in order to significantly reduce the prevalence of the disease and eventually eradicate the infection [13]. It has been demonstrated that the most efficient oral vaccine bait deployment strategy in this area consists in using selective piglet feeders and deploying vaccine baits both at the start (late June/early July) and the end of summer (late August/early September), in order to gain access to a high proportion of early and late born wild boar piglets respectively, avoiding the hottest period from late July to early August [14,15]. However, with this strategy of a double delivery in summer, it would be probable that some individuals actually gain access to vaccine baits twice in a season. This possibility of some wild boar ingesting vaccine baits in both vaccination campaigns raises the question of the effects of re-vaccination in wild boar. In human beings, revaccination with BCG confers only a modest advantage over single vaccination [16,17]. Among cattle and wildlife MTC hosts, there are controversial studies regarding the use of a single dose or the need of a booster dose of the vaccine to achieve a better vaccination protocol (Table 1). Re-vaccination has the potential to be counter-productive, and such risks needed to be assessed in wild boar as an integral part of vaccine safety. Although adverse effects of re-vaccination or over-dosage of BCG in wild boar were not expected, we were interested in assessing the effects of re-vaccination with a 52 day interval imitating field conditions.

**Table 1:** BCG vaccination studies comparing the use of both one single dose or repeated doses of the vaccine in cattle and wildlife

	**Dose**	**Interval**	**Route**	**Reference**	**Effect of boosting**
**Red deer (*Cervus elaphus*)**	2 doses of 2.5×10^6^ cfu BCG Pasteur	8 weeks	Subcutaneous	[5]	Two doses of vaccine were superior to one dose producing protection against infection.
**Red deer (*Cervus elaphus*)**	2 doses of 2×10^6^ cfu BCG Pasteur	6 weeks	Subcutaneous	[18]	Single-dose vaccines protected against disease. Boosting was required to protect against infection.
**White-tailed deer (*Odocoileus virginianus*)**	2 doses of 10^7^ cfu BCG Pasteur	6 weeks	Subcutaneous	[19]	Pathology scores were lower in deer receiving 2 doses of *M. bovis* BCG compared to unvaccinated ones or those who received a single dose.
**Brush-tailed possum (*Trichosurus vulpecula*)**	12 doses of 1×10^8^; 2 doses of 1×10^8^ cfu BCG Pasteur	1 Week (12 doses); 6 weeks (2 doses)	Intranasal aerosol and conjuntival instillation	[20]	The group vaccinated 12 times showed the greatest level of protection. Revaccination after a period of 6 weeks had no beneficial and no deleterious effects as compared to the protection induced by a single dose of vaccine.
**Cattle**	2 doses of 10^6^ CFU BCG Pasteur	6 weeks	Subcutaneous	[21]	Significantly less protection than those vaccinated only once. Revaccination reduced the level of protection induced by a single vaccination.

The aim of this study was to assess the response of wild boar to re-vaccination with BCG and to subsequent challenge with an *M. bovis* field strain, under experimental conditions. Based on current knowledge, we expected a protective response similar to those recorded in previous single dose BCG vaccination experiments [2,22].

## Methods

### Experiment design

Seventeen 3-4-month-old wild boar piglets were bought in a commercial farm known to be free of mycobacterial lesions at slaughter and with a fully negative ELISA test [23]. The animals were housed in class III bio-containment facilities with ad libitum food and water. Wild boar piglets were randomly assigned to treatment (BCG vaccinated animals) or control groups (unvaccinated animals). The *M. bovis* BCG Danish reference strain (CCUG 27863) was cultured on Coletsos medium (Biomerieux, France), and prepared as described for previous experiments [22]. Vaccine containing 10^6^ cfu was administered orally in baits designed for wild boar piglets [24]. For the challenge, 5 ml of a suspension containing 10^5^ colony forming units (cfu) of an *M. bovis* field strain were administered by the oropharyngeal route as described in previous experiments [2,22]. The isolate used for challenge was originally isolated from a naturally infected wild boar and identified as *M. bovis* spoligotype profile SB0339 according to the *M. bovis* Spoligotype Database website (http://www.mbovis.org).

The animals were handled five times during the experiment, including vaccination (T0, day 1), re-vaccination 52 days after the first vaccination (T1, day 52), challenge 74 days after re-vaccination (T2, day 126), one blood sampling two months after challenge (T3, day 185) and necropsy four months after challenge (T4, day 255). Handling procedures and sampling frequency were designed to reduce stress and health risks for subjects, according to European (86/609) and Spanish laws (R.D. 223/1988, R.D. 1021/2005). The protocol was approved by the Committee on the Ethics of Animal Experiments of the Regional Agriculture Authority (Diputación Foral de Vizcaya, Permit Number: BFA10.373 (27/19/2010)).

### Sampling, pathology and microbiology

Blood samples were collected at T0-T4 time points for RNA extraction from PBMC and serum preparation. Animals were anesthetized by intramuscular injection of Zoletil (Virbac, Esplugues de Llobregat, Spain) and euthanized by captive bolt. At necropsy, all wild boar were carefully inspected for the presence of macroscopic TB-compatible lesions. Samples for culture were immediately processed and copies frozen at -80°C for mRNA isolation. TB-compatible lesions were classified based on lesion distribution and lesion intensity, and scored as previously described [2]. Briefly, lymph nodes and the oropharyngeal tonsils were scored as 0 (No visible lesion), 1 (1–2 small (<1 cm) caseous foci), 2 (Several small foci), 3 (Same and at least one lesion >1 cm) or 4 (Diffusely affected); lung lobes were scored as 0 (No visible lesion), 1 (Few small lesions), 2 (Numerous or clustered small lesions with some coalescence), 3 (Densely clustered small lesions), 4 (Same and at least one large lesion) or 5 (Two or more large lesions). Visceral organ lesions were scored as 0 (No visible lesion), 1 (1–2 mm foci scattered throughout organ) or 2 (5–10 mm diameter clusters of 1–2 mm foci or single focus >1 cm diameter). Lymphoid tissues and samples of lung tissue were cultured for mycobacteria and scored (total number of culture-positive samples) as described in [22]. All isolates were spoligotyped in order to confirm the strain [25]. Tonsil samples were flash frozen and stored in liquid N until used for gene expression studies.

### Serology and interferon tests

Serum samples were tested for anti-PPD immunoglobulin G (IgG) antibodies by means of an in-house ELISA using bovine tuberculin purified protein derivative (bovine PPD; CZ Veterinaria SL, Porriño, Lugo, Spain) as antigen and protein G horseradish peroxidase (Sigma-Aldrich, Madrid, Spain) as a conjugate applying the protocol described by Boadella et al. [23]. Serum samples were also tested by the DPP technology developed by Chembio Diagnostic Systems, Inc. using selected *M. bovis* antigens. Briefly, the presence and intensity of either of the 2 separate test lines (T1, MPB83 antigen; T2, CFP10/ESAT-6 fusion protein) were evaluated by a DPP optical reader (in relative light units, RLU) [23]. Blood samples taken at times 2, 3 and 4 were also used for detection of the IFN-gamma response in presence of avian and bovine PPD, as described previously [22].

### RNA isolation and real time RT-PCR

Total RNA was extracted from wild boar PBMC and tonsils using TRI reagent (Sigma, Madrid, Spain) following manufacturer’s recommendations. RNA was used for real-time RT-PCR analysis of mRNA levels of selected genes in individual samples. Selected genes are involved in innate immunity (complement component 3, *C3* and interleukin 1-beta, *IL-1b*) and methylmalonyl CoA mutase, *MUT*. Real-time RT-PCR was performed with gene-specific primers (*C3*, SsC3-L: acaaattgacccagcgtagg and SsC3-R: gcacgtccttgctgtactga; *IL-1b*, SsIL1beta-L: ccaaagagggacatggagaa and SsIL1beta-R: ttatatcttggcggcctttg; *MUT*, SsMUT-L: gtttgccaacggtgaaaagt and SsMUT-R: aatgagcttcaaggcagcat) using the One-Step RT-PCR Kit with SYBR Green and the CFX thermal cycler (Bio-Rad, Hercules, CA, USA) following manufacturer’s recommendations. Control reactions were performed using the same procedures, but without RT to monitor DNA contamination in the RNA preparations and without RNA added to monitor contamination of the PCR reaction. A dissociation curve was run at the end of RT-PCR reaction to ensure that only one amplicon was formed and that the amplicon denatured consistently at the same temperature range for every sample [26]. The mRNA values were normalized against *S. scrofa* cyclophilin (SsCyclophilin-L: agcactggggagaaaggatt and SsCyclophilin-R: cttggcagtgcaaatgaaaa), β-actin (Ss-BactinF: ggacctgaccgactacctca and Ss-BactinR: ggcagctcgtagctcttcat) and GAPHD (Ss-GAPHDF: gtcggttgtggatctgacct and Ss-GAPHDR: agcttgacgaagtggtcgtt) using the genNorm ddCT method [27].

## Results

**Figure 1:**
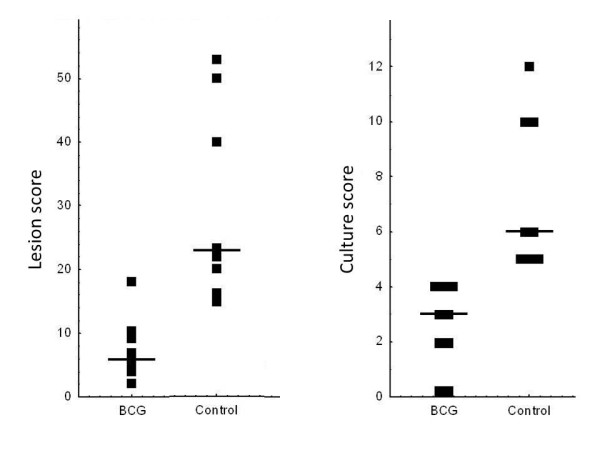
**Wild boar TB lesion scores (left) and culture scores (right) at necropsy.** The solid lines show the median values.

No clinical signs such as wasting or cough were recorded during the experiment. Figure 1 presents the pathology scores and total culture scores. BCG re-vaccinated wild boar showed reductions of 75.8% and 66.9%, respectively, as compared to controls (U tests; Z > 3.2, p < 0.01). Two BCG re-vaccinated wild boar had no positive culture. The mean (±SE) thorax culture scores were 0.22 (±0.3) for vaccinated and 2.25 (±0.3) for control wild boar (90.2% reduction; *U* test; Z = 3.1, p < 0.01). Only one of nine vaccinated wild boar had a culture-confirmed lung infection, as compared to seven of eight controls (Fisher’s test; p = 0.003).

**Figure 2:**
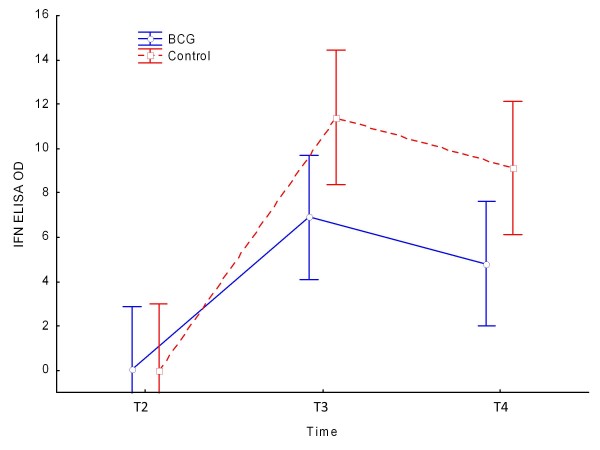
**Mean optical density (OD) readings of the gamma interferon ELISA in BCG re-vaccinated (solid line) and control (dashed line) wild boar.** Vertical bars represent 95% confidence intervals.

Serum antibody levels were highly variable and did not differ significantly between BCG re-vaccinated wild boar and controls (ANOVA; Treatment effect F_1, 45_ < 2.71, p > 0.05). Gamma IFN levels differed significantly between BCG re-vaccinated wild boar and controls (ANOVA; Treatment effect F_1, 39_ = 6.08, p < 0.05). The gamma IFN response to bPPD was undetectable at T2. A peak was recorded at T3 both in BCG re-vaccinated wild boar (mean OD ± SE 6.9 ± 1.7) and in controls (11.4 ± 1.8), slightly declining in both groups thereafter (Figure 2).

**Figure 3:**
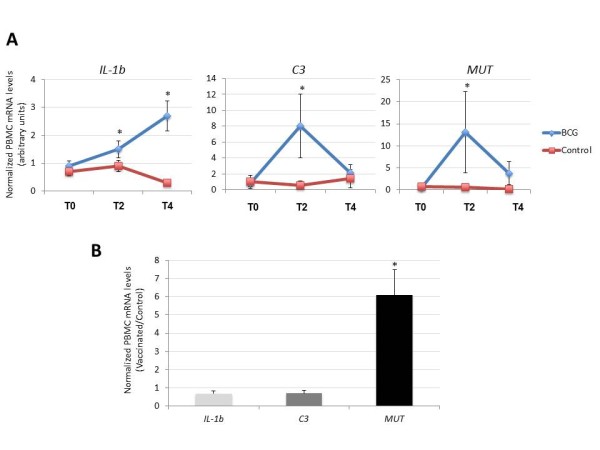
**Gene expression in response to BCG vaccination in wild boar.** The mRNA levels of selected genes were analyzed by real-time RT-PCR in **(A)** PBMC of vaccinated and control wild boar collected at T0 (before vaccination), T2 (before challenge) and T4 (at necropsy) or **(B)** tonsils at T4. The mRNA levels were normalized against *S. scrofa* cyclophilin, β-actin and GAPHD and normalized Ct values were represented as Ave + S.D. in arbitrary units and compared between groups by Student´s *t*-test with unequal variance (*P ≤ 0.05).

The mRNA levels for IL-1b, C3 and MUT were significantly higher in vaccinated wild boar when compared to controls at T2 and decreased after mycobacterial challenge (Figure 3A). Only IL-1b mRNA levels remained higher in vaccinated animals when compared to controls until the end of the experiments (Figure 3A). In tonsils, only MUT mRNA levels were significantly higher in vaccinated than in control animals at the end of the experiment (Figure 3B).

## Discussion

This experiment confirmed that oral BCG re-vaccination of wild boar induces a strong protective response against challenge with an *M. bovis* field strain. This response includes lower lesion and culture scores; lower gamma IFN levels and higher IL-1b, C3 and MUT mRNA levels in vaccinated wild boar.

The main difference between this experiment and the previous vaccine and challenge trials in wild boar was re-vaccination. Although no single vaccination controls were included, the protective response recorded after re-vaccination (76% lesion score, 67% culture score), was higher than those recorded in two previous single dose BCG vaccination experiments, i.e. 56% reduction in lesion score, 50% in culture score [2]; 52% reduction in lesion score, 9% in culture score [22]. This animal experiment was run along with another one testing a new, heat-inactivated vaccine, and the number of available experimental wild boar and the available housing space did not allow including a single-vaccination BCG group. Serum antibody and gamma IFN responses were essentially similar to those recorded in these single vaccination experiments. These levels of protection were better than those documented for BCG-Pasteur re-vaccinated African buffaloes (*Syncerus caffer*), in which revaccination gave not differences in the protection for vaccinated animals compared to unvaccinated animals [4]. In ferrets (*Mustela furo*), two doses of 5×10^8^ cfu of BCG-Pasteur given orally in a 4 week interval produced a significant reduction in pathology after oral challenge with virulent *M. bovis*, decreasing the mean bacterial count for retropharyngeal LN tissue of culture- positive vaccinated animals to 70% [7].

It is known that factors such as the variation of the period between the administration of the two doses of the vaccine could affect the protective efficacy of the vaccination. For example, the reduction of this period from 8 to 4 weeks in vaccinated red deer did not affect the protection induced by the vaccine, whereas increasing this period to 43 weeks largely ablated protection [28]. In our study, wild boar vaccination took place 52 days after the first vaccination (between 7-8 weeks), which imitates field conditions.

Another important factor that could affect the protection of the vaccine is the dose. Low and medium booster doses (10^4^-10^7^ cfu; 6-8 weeks interval) of BCG-Pasteur produced significant protection against infection and disease in vaccinated red deer, but higher booster doses (5×10^8^cfu) provided protection against disease but not against infection [5,18]. In our case, a dose of 10^6^ cfu was used, again imitating field protocols.

Molecular characterization of host-pathogen interactions identified wild boar genes such as MUT, C3 and other innate and adaptive immune response genes involved in resistance to mycobacterial infection [29-33]. As in previous experiments, C3 and MUT mRNA levels rose after vaccination and decreased after challenge [22]. The up-regulation of genes encoding pro-inflammatory cytokines such as pro-IL-1b probably stimulates the production of C3 which may contribute to the protective response to BCG vaccination in wild boar.

## Conclusions

Oral re-vaccination of wild boar with BCG yields a strong protective response against challenge with a field strain. One important conclusion of this study is that re-vaccination of wild boar with BCG is not counterproductive as suggested for cattle [34]. This finding is relevant given that wild boar TB is increasingly reported [35,36] and that re-vaccination is likely to happen in the ongoing field vaccination trials.

## Competing interests

The authors declare that they have no competing interests.

## Authors’ contributions

BB-B, JMG, AA, LD, RJ, JdlF and CG: contributed to the conception, design, and data collection, laboratory work, drafting and writing of the manuscript. IAS, MB, KPL, RCG and VM: contributed to laboratory work, data analysis and drafting of the manuscript. All authors have read and approved the final manuscript.

## References

[B1] Calmette A La vaccination preventive contra la tuberculose 1927 Paris: Masson

[B2] Ballesteros C Garrido JM Vicente J Romero B Galindo RC Minguijón E Villar M Martín-Hernando MP Sevilla I Juste R Aranaz A de la Fuente J Gortázar C First data on Eurasian wild boar response to oral immunization with BCG and challenge with a Mycobacterium bovis field strain Vaccine 2009 27 6662 6668 19747578 10.1016/j.vaccine.2009.08.095

[B3] Corner LAL Costello E O'Meara D Lesellier S Aldwell FE Singh M Hewinson RG Chambers MA Gormley E Oral vaccination of badgers (Meles meles) with BCG and protective immunity against endobronchial challenge with Mycobacterium bovis Vaccine 2010 28 6265 6272 20637774 10.1016/j.vaccine.2010.06.120

[B4] de Klerk LM Michel AL Bengis RG Kriek NPJ Godfroid J BCG vaccination failed to protect yearling African buffaloes (Syncerus caffer) against experimental intratonsilar challenge with Mycobacterium bovis Vet Immunol Immunopathol 2010 137 84 92 20684850 10.1016/j.vetimm.2010.04.013

[B5] Griffin JFT Mackintosh CG Slobbe L Thomson AJ Buchan GS Vaccine protocols to optimise the protective efficacy of BCG Tuber Lung Dis 1999 79 135 143 10656112 10.1054/tuld.1998.0202

[B6] Nol P Palmer MV Waters WR Aldwell FE Buddle BM Triantis JM Linke LM Phillips GE Thacker TC Rhyan JC Dunbar MR Salman MD Efficacy of oral and parenteral routes of Mycobacterium bovis bacilli Calmette-Guerin vaccination against experimental bovine tuberculosis in white-tailed deer (Odocoileus virginianus): A feasibility study J Wildl Dis 2008 44 247 259 18436658 10.7589/0090-3558-44.2.247

[B7] Qureshi T Labes RE Cross ML Griffin JFT Mackintosh CG Partial protection against oral challenge with Mycobacterium bovis in ferrets (Mustela furo) following oral vaccination with BCG Int J Tuberc Lung Dis 1999 3 1025 1033 10587325

[B8] Tompkins DM Ramsey DSL Cross ML Aldwell FE De Lisle GW Buddle BM Oral vaccination reduces the incidence of tuberculosis in free-living brushtail possums Proc R Soc Lond B Biol Sci 2009 276 2987 2995 10.1098/rspb.2009.0414 PMC2817216 19493904

[B9] Gormley E Corner LAL Control strategies for wildlife tuberculosis in Ireland Transbound Emerg Dis 2013 60 128 135 24171858 10.1111/tbed.12095

[B10] Gortázar C Delahay RJ McDonald RA Boadella M Wilson GJ Gavier-Widen D Acevedo P The status of tuberculosis in European wild mammals Mammal Rev 2012 42 193 206

[B11] Gortazar C Vicente J Boadella M Ballesteros C Galindo RC Garrido J Aranaz A de la Fuente J Progress in the control of bovine tuberculosis in Spanish wildlife Vet Microbiol 2011 151 170 178 21440387 10.1016/j.vetmic.2011.02.041

[B12] Naranjo V Gortazar C Vicente J de la Fuente J Evidence of the role of European wild boar as a reservoir of Mycobacterium tuberculosis complex Vet Microbiol 2008 127 1 9 18023299 10.1016/j.vetmic.2007.10.002

[B13] Anderson LG Gortázar C Vicente J Hutchings MR White PCL Modelling the effectiveness of vaccination in controlling bovine tuberculosis in wild boar Wildlife Res 2013 40 367 376

[B14] Ballesteros C Vicente J Carrasco-García R Mateo R de la Fuente J Gortázar C Specificity and success of oral-bait delivery to Eurasian wild boar in Mediterranean woodland habitats E J Wildlife Res 2011 57 749 757

[B15] Beltrán-Beck B Ballesteros C Vicente J De La Fuente J Gortázar C Progress in oral vaccination against tuberculosis in its main wildlife reservoir in Iberia, the Eurasian wild boar Vet Med Int 2012 doi:10.1155/2012/978501 10.1155/2012/978501 PMC3400400 22848869

[B16] Barreto ML Pereira SM Pilger D Cruz AA Cunha SS Sant'Anna C Ichihara MY Genser B Rodrigues LC Evidence of an effect of BCG revaccination on incidence of tuberculosis in school-aged children in Brazil: Second report of the BCG-REVAC cluster-randomised trial Vaccine 2011 29 4875 4877 21616115 10.1016/j.vaccine.2011.05.023

[B17] Whelan KT Pathan AA Sander CR Fletcher HA Poulton I Alder NC Hill AVS McShane H Safety and immunogenicity of boosting BCG vaccinated subjects with BCG: Comparison with boosting with a new TB vaccine, MVA85A PLoS One 2009 4 e5934 doi:10.1371/journal.pone.0005934 19529780 10.1371/journal.pone.0005934 PMC2694271

[B18] Griffin JF Veterinary tuberculosis vaccine development Clin Infect Dis 2000 30 S223 S228 10875788 10.1086/313865

[B19] Palmer MV Thacker TC Waters WR Vaccination of white-tailed deer (Odocoileus virginianus) with Mycobacterium bovis bacillus Calmette Guerın Vaccine 2007 25 6589 6597 17688976 10.1016/j.vaccine.2007.06.056

[B20] Corner LAL Buddle BM Pfeiffer DU Morris RS Vaccination of the brushtail possum (Trichosurus vulpecula) against Mycobacterium bovis infection with bacille Calmette-Guerin: the response to multiple doses Vet Microbiol 2002 84 327 336 11750141 10.1016/s0378-1135(01)00461-8

[B21] Buddle BM Wedlock DN Parlane NA Corner LAL de Lisle GW Skinner MA Revaccination of Neonatal Calves with Mycobacterium bovis BCG reduces the level of protection against bovine tuberculosis induced by a single vaccination Infect Immun 2003 71 6411 6419 14573662 10.1128/IAI.71.11.6411-6419.2003 PMC219550

[B22] Garrido JM Sevilla IA Beltrán-Beck B Minguijón E Ballesteros C Galindo RC Boadella M Lyashchenko KP Romero B Geijo MV Ruiz-Fons F Aranaz A Juste RA Vicente J de la Fuente J Gortázar C Protection against Tuberculosis in Eurasian Wild Boar Vaccinated with Heat-inactivated Mycobacterium bovis PLoS One 2011 6 e24905 doi:10.1371/journal.pone.0024905 21935486 10.1371/journal.pone.0024905 PMC3173485

[B23] Boadella M Lyashchenko K Greenwald R Esfandiari J Jaroso R Carta T Garrido JM Vicente J de la Fuente J Gortazar C Serological tests for detecting antibodies against Mycobacterium bovis and Mycobacterium avium subspecies paratuberculosis in Eurasian wild boar (Sus scrofa scrofa) J Vet Diagn Invest 2011 23 77 83 21217031 10.1177/104063871102300111

[B24] Ballesteros C Gortázar C Canales M Vicente J Lasagna A Gamarra JA Carrasco-García R Fuente J Evaluation of baits for oral vaccination of European wild boar piglets Res Vet Sci 2009 86 388 393 18950820 10.1016/j.rvsc.2008.09.003

[B25] Kamerbeek J Schouls L Kolk A van Agterveld M van Soolingen D Kuijper S Bunschoten A Molhuizen H Shaw R Goyal M van Embden J Simultaneous detection and strain differentiation of Mycobacterium tuberculosis for diagnosis and epidemiology J Clin Microbiol 1997 35 907 914 9157152 10.1128/jcm.35.4.907-914.1997 PMC229700

[B26] Ririe KM Rasmussen RP Wittwer CT Product differentiation by analysis of DNA melting curves during the polymerase chain reaction Anal Biochem 1997 245 154 160 9056205 10.1006/abio.1996.9916

[B27] Livak KJ Schmittgen TD Analysis of relative gene expression data using real-time quantitative PCR and the 2 -ΔΔCT method Methods 2001 25 402 408 11846609 10.1006/meth.2001.1262

[B28] Griffin JF Mackintosh CG Rodgers CR Factors influencing the protective efficacy of a BCG homologous prime-boost vaccination regime against tuberculosis Vaccine 2006 24 835 845 16098638 10.1016/j.vaccine.2005.07.033

[B29] Naranjo V Ayoubi P Vicente J Ruiz-Fons F Gortazar C Kocan KM de la Fuente J Characterization of selected genes upregulated in non-tuberculous European wild boar as possible correlates of resistance to Mycobacterium bovis infection Vet Microbiol 2006 116 224 231 16672181 10.1016/j.vetmic.2006.03.013

[B30] Naranjo V Höfle U Vicente J Martin MP Ruiz-Fons F Gortazar C Kocan KM de la Fuente J Genes differentially expressed in oropharyngeal tonsils and mandibular lymph nodes of tuberculous and non-tuberculous European wild boars naturally exposed to Mycobacterium bovis FEMS Immunol Med Microbiol 2006 98 77 85 10.1111/j.1574-695X.2005.00035.x 16487312

[B31] Naranjo V Villar M Martín-Hernando MP Vidal D Höfle U Gortazar C Kocan KM Vázquez J de la Fuente J Proteomic and transcriptomic analyses of differential stress/inflammatory responses in mandibular lymph nodes and oropharyngeal tonsils of European wild boars naturally infected with Mycobacterium bovis Proteomics 2007 7 220 231 17163576 10.1002/pmic.200600527

[B32] Galindo RC Ayoubi P Naranjo V Gortazar C de la Fuente J Gene expression profiles of European wild boar naturally infected with Mycobacterium bovis Vet Immunol Immunopathol 2009 129 119 125 19131115 10.1016/j.vetimm.2008.12.012

[B33] de la Lastra JM P Galindo RC Gortázar C Ruiz-Fons F Aranaz A de la Fuente J Expression of immunoregulatory genes in peripheral blood mononuclear cells of European wild boar immunized with BCG Vet Microbiol 2009 134 334 339 19095381 10.1016/j.vetmic.2008.08.026

[B34] Buddle BM Pollock JM Skinner MA Wedlock DN Development of vaccines to control bovine tuberculosis in cattle and relationship to vaccine development for other intracellular pathogens Int J Parasitol 2003 33 555 566 12782055 10.1016/s0020-7519(03)00060-2

[B35] Muñoz-Mendoza M Marreros N Boadella M Gortázar C Menéndez S de Juan L Bezos J Romero B Copano MF Amado J Sáez JL Mourelo J Balseiro A Wild boar tuberculosis in Iberian Atlantic Spain: a different picture from Mediterranean habitats BMC Vet Res 2013 9 176 24010539 10.1186/1746-6148-9-176 PMC3844463

[B36] Richomme C Boadella M Courcoul A Durand B Drapeau A Corde Y Hars J Payne A Fediaevsky A Boschiroli ML Exposure of Wild Boar to Mycobacterium tuberculosis Complex in France since 2000 Is Consistent with the Distribution of Bovine Tuberculosis Outbreaks in Cattle PLoS One 2013 8 e77842 doi:10.1371/journal.pone.0077842 24167584 10.1371/journal.pone.0077842 PMC3805591

